# IslandViewer 3: more flexible, interactive genomic island discovery, visualization and analysis

**DOI:** 10.1093/nar/gkv401

**Published:** 2015-04-27

**Authors:** Bhavjinder K. Dhillon, Matthew R. Laird, Julie A. Shay, Geoffrey L. Winsor, Raymond Lo, Fazmin Nizam, Sheldon K. Pereira, Nicholas Waglechner, Andrew G. McArthur, Morgan G.I. Langille, Fiona S.L. Brinkman

**Affiliations:** 1 Department of Molecular Biology and Biochemistry, Simon Fraser University, Burnaby, BC V5A 1S6, Canada; 2 M.G. DeGroote Institute for Infectious Disease Research, Department of Biochemistry and Biomedical Sciences, DeGroote School of Medicine, McMaster University, Hamilton, ON L8S 4K1, Canada; 3 Department of Pharmacology, Dalhousie University, Halifax, NS B3H 4R2, Canada

## Abstract

IslandViewer (http://pathogenomics.sfu.ca/islandviewer) is a widely used web-based resource for the prediction and analysis of genomic islands (GIs) in bacterial and archaeal genomes. GIs are clusters of genes of probable horizontal origin, and are of high interest since they disproportionately encode genes involved in medically and environmentally important adaptations, including antimicrobial resistance and virulence. We now report a major new release of IslandViewer, since the last release in 2013. IslandViewer 3 incorporates a completely new genome visualization tool, IslandPlot, enabling for the first time interactive genome analysis and gene search capabilities using synchronized circular, horizontal and vertical genome views. In addition, more curated virulence factors and antimicrobial resistance genes have been incorporated, and homologs of these genes identified in closely related genomes using strict filters. Pathogen-associated genes have been re-calculated for all pre-computed complete genomes. For user-uploaded genomes to be analysed, IslandViewer 3 can also now handle incomplete genomes, with an improved queuing system on compute nodes to handle user demand. Overall, IslandViewer 3 represents a significant new version of this GI analysis software, with features that may make it more broadly useful for general microbial genome analysis and visualization.

## INTRODUCTION

Genomic islands (GIs) are commonly defined as clusters of genes of probable horizontal origin >8 kb in size in bacterial and archaeal genomes (1). They play a significant role in the genome evolution of such microbes, encoding genes involved in notable adaptations of medical or environmental interest (1–3). Many virulence factor and/or antimicrobial resistance genes are shared and acquired via GIs (4) and genes such as virulence factors have been shown to be disproportionately associated with these GI regions (5). For the computational detection of GIs, IslandViewer is a freely-available web-based resource that incorporates three of the most accurate GI prediction methods: IslandPick, IslandPath-DIMOB and SIGI-HMM (6–9). These methods predict GIs based on different, complementary features. IslandPick uses a comparative genomics-based approach, identifying unique regions by comparing a user-specified genome against closely related genomes (that may also be flexibly specified). IslandPath-DIMOB and SIGI-HMM are sequence composition approaches, with IslandPath-DIMOB identifying islands with dinucleotide bias and the presence of an associated mobility gene (integrases, transposases, etc.), while SIGI-HMM identifies codon usage bias with a hidden Markov model approach. User-uploaded custom genomes may be analysed, plus pre-computed analyses have been made available for all NCBI complete microbial genomes. In previous versions of IslandViewer, a basic circular genome visualizer has allowed users to view GIs, with links to a table providing annotations of the genes in the predicted GI regions. Curated virulence factors and antimicrobial resistance genes were provided on a subset of pre-computed genomes, along with a previously assessed analysis of pathogen-associated genes (5,10–12). Now, since the last update of IslandViewer published in 2013 (12), IslandViewer has been completely re-written and boasts a new interactive, flexible genome visualization tool, IslandPlot, with synchronized circular, horizontal and vertical genome views, gene searching capabilities, expanded virulence, antimicrobial resistance and pathogen-associated gene annotations, annotation of close homologs of virulence and antimicrobial resistance genes using strict criteria, and additional back-end web server upgrades for improved queuing and handling of custom genomes requiring analysis. By popular request, incomplete genomes may now also be analysed. Together, these changes are released as IslandViewer 3, the most comprehensive and flexible GI analysis web server available to date.

## INNOVATIVE, INTERACTIVE GENOME VISUALIZATION AND SEARCHING

For IslandViewer 3, IslandPlot has been developed as a new genome visualization library based on the D3 javascript library (http://www.d3js.org). With this new tool, interactive circular, horizontal and vertical genome views are provided in IslandViewer for both user-uploaded and pre-computed genome analyses. Importantly, IslandPlot is able to generate visualizations dynamically, eliminating the need to store pre-computed images for every permutation of information to display, as was done previously. This greatly improves IslandViewer's ability to handle the increasing number of bacterial and archaeal genomes while minimizing storage requirements and provides a richer, more interactive genome browsing experience. Within the three separate views, GI predictions are shown, broken down by prediction method, along with annotations of virulence factors, antimicrobial resistance genes and pathogen-associated genes using previously accepted methods (10–13). Users can specify which GI prediction method and annotations to display, and can select regions in the circular view to zoom in or out, which updates the horizontal and vertical genome on the selected region. While the horizontal viewer provides a more detailed visual representation of a selected region of the genome, the vertical viewer provides text descriptions of the genes and gene products located within the genomic region of interest. Both horizontal and vertical viewers have their own zoom/navigation features (using a mouse scroll wheel, for example over the horizontal view). For pre-computed genome analyses, both viewers provide links to the NCBI for any selected gene, or in the case of virulence and antimicrobial resistance gene annotations, links are provided to the source of the annotation for more information. A side-by-side comparison of two genomes is also supported by IslandPlot. Additionally, as users navigate through a genome of interest, a page can now be ‘saved’ for linking back to selected regions and zoom levels using a unique URL. Source code for IslandPlot (also known at time of publication as GenomeD3Plot) is freely available through GitHub at https://github.com/brinkmanlab/GenomeD3Plot.

**Figure 1.**
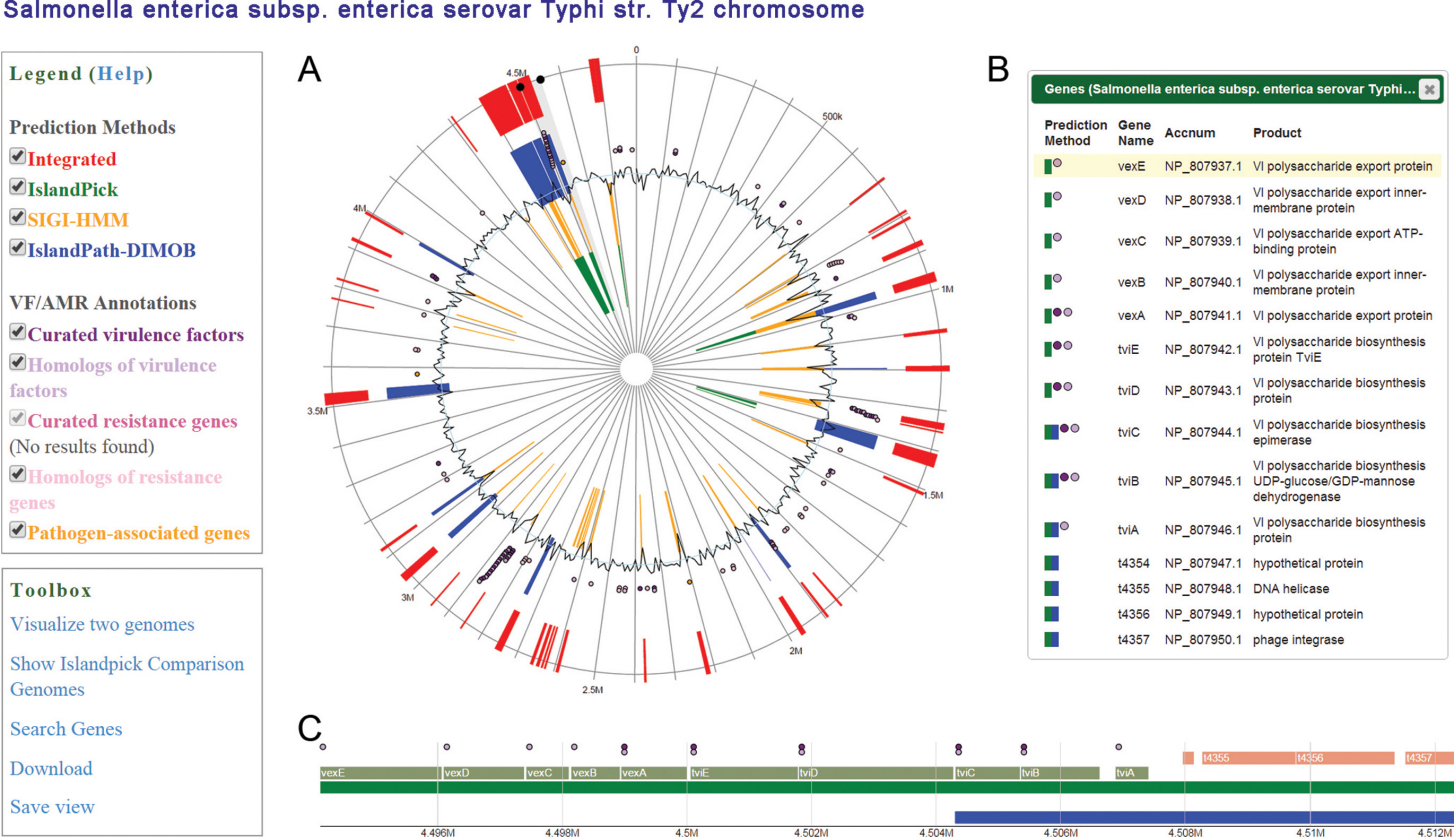
IslandViewer 3 results page for *Salmonella enterica* subsp. *enterica* serovar Typhi str. Ty2 (accession number NC_004631.1). Panel (**A**) is the circular genome view, panel (**B**) is the vertical view with text descriptions of genes and gene products and panel (**C**) is the horizontal view to enable users to zoom in/out of regions of interest. Within each panel, GI predictions are represented as blocks (integrated results in red, IslandPick in green, SIGI-HMM in orange and IslandPath-DIMOB in blue), virulence genes (purple for curated, light purple for homologs), antimicrobial resistance genes (pink for curated, light pink for homologs) and pathogen-associated genes (orange) as circular glyphs. The legend can be used to turn individual items on/off in the circular view. Users can click on any island or double-click on any other region of panel (A), which will then zoom-in to those regions in panels (B) and (C) for more details. New in the toolbox, users can now search for a particular gene of interest and save a customized view.

Because of the interactive nature of IslandPlot, IslandViewer 3 also allows users to search for particular genes of interest within a genome, and will highlight the genes of interest in each view. This function allows users to better navigate through a genome of interest. Figure 1 shows the IslandPlot visualizations for *Salmonella enterica* subsp. *enterica* serovar Typhi str. Ty2 after searching for a known virulence factor, *vexE*.This gene is focused in all views and highlighted in the vertical panel to easily evaluate the GI predictions and annotations of *vexE* and its neighboring genes. This IslandPlot viewer and associated gene search functionality can serve as a broader search tool to study a genome of interest at various levels of detail, including virulence, resistance or pathogen-associated genes.

## EXPANDED VIRULENCE FACTOR AND ANTIMICROBIAL RESISTANCE GENE ANNOTATIONS

### Antimicrobial resistance gene annotations

In the previous IslandViewer update, 956 antimicrobial resistance gene annotations were incorporated from the Antibiotic Resistance Genes Database (ARDB) (11). To further improve resistance gene annotations, all proteins in IslandViewer 3 at the time of publication have been analyzed using the most precise version of the previously published Resistance Gene Identifier (RGI) method for identifying genes involved in antimicrobial resistance (13). The RGI uses a BLASTp search against the set of curated resistance genes in the Comprehensive Antibiotic Resistance Database (CARD; 7 November 2014 set of curated data) with precise filtering criterion based on different types of resistance classes. This sophisticated RGI analysis also includes identification of single nucleotide variations (SNVs) that confer resistance (13). The antimicrobial resistance gene annotations from ARDB have been curated and incorporated into CARD, thus, these new annotations have replaced the previous ARDB annotations in IslandViewer. Exact matches to curated antimicrobial resistance genes are denoted as curated resistance genes and colored in pink in IslandViewer 3. Any hits that were found within the strict criterion are denoted as homologs and are displayed in light pink. Through this analysis, antimicrobial resistance gene annotations for pre-computed genomes have been greatly expanded from 956 to 28 911 resistance genes, providing an overview of predicted molecular antimicrobial resistance profiles for 589 distinct genera available to date in IslandViewer.

### Pathogen-associated gene annotations

The previously published pathogen-associated genes analysis was updated using the same methodology as outlined by Ho Sui *et al*. in 2009 (5). The original analysis was performed on 631 genomes, of which 298 were pathogens. An update of this analysis to include every genome completely sequenced since then (and available through NCBI) required us to manually curated 2794 genomes as either from a pathogen or non-pathogen, using the same criteria accepted in the previous analysis. In total, 1277 pathogen and 1517 non-pathogen genomes were compared, to determine the set of genes currently specific for pathogen genomes using set criteria, termed pathogen-associated genes. These pathogen-associated genes have been shown to be disproportionately involved in more ‘offensive’ virulence roles such as invasion into a host, type III/IV secretion systems, or toxins, rather than defence roles. Such genes are of interest since they may represent novel virulence factors under certain conditions. After this updated analysis, 18 919 pathogen-associated genes (found in three or more distinct genera) were identified and annotated in IslandViewer 3. All results from this analysis are also available for reference at http://pathogenomics.sfu.ca/pathogen-associated/2014.

### Expanded virulence factor gene annotations

Virulence factors have been demonstrated to be associated with GIs (5), which represent a subclass of GIs known as pathogenicity islands (PAIs) (14,15). To aid identification of PAIs within GI predictions, an expanded virulence factor annotation was completed. More than 1600 curated virulence factors were annotated in the last release of IslandViewer using the VFDB (10). Since then, over 8000 additional curated virulence factor gene annotations have been collected from the expanded Virulence Factor Database (VFDB) (10), PATRIC (16) and Victor's virulence factors (http://www.phidias.us/victors/) and mapped to their corresponding proteins in IslandViewer 3. Only a subset of virulence factor annotations from PATRIC, those with curated links to literature, were incorporated. These annotations are displayed in purple in the genome visualizations.

However, such curated virulence factors still only cover a limited number of genomes and as the number of very closely related genomes sequenced increases, it is clear that many of these curated virulence factors should also be annotated in highly similar genomes of the same species or serovar. To address this issue, a very conservative reciprocal best blast hit (RBBH) approach was used to identify homologs (essentially probable orthologs) of curated virulence factor genes using strict criteria: the virulence factor annotation transfer was only permitted if the gene occurred within the same species, plus the CVTree (17) distance between the genomes being compared was <0.3 (to ensure that annotation transfer did not occur even within a species if the genomes were more divergent). Additional filters were placed specifically upon genera with notable phenotypic variability within the species, e.g. *Salmonella* and *Escherichia* genera, such that annotation transfer was only permitted between genomes from the same serovar or strain for such species. Annotations were also only transferred if the RBBH BLASTp *e*-value was lower than 1e–10, plus the sequences shared ≥90% sequence identity, plus the BLAST hit (high scoring segment pair) also covered at least 80% of the query sequence length. These very stringent criteria were selected in order to maximize precision/specificity for the annotation of virulence factor gene homologs at the expense of recall/sensitivity, since we wanted to ensure annotations would be most likely correct, at the expense of missing some. Even though this criteria tends to identify orthologs by widely accepted RBBH criteria, we err on the side of just calling them homologs, and annotate them with a different lighter purple color in the genome visualization, to highlight that they are not confirmed, curated virulence factors. This approach was very successful, allowing us to annotate an additional 39 441 virulence gene homologs in 485 genomes, covering 37 distinct pathogen genera. With this expanded dataset, users can view and explore the presence/absence of PAIs in many more genomes than previously, including very closely related genomes from different strains of a species which clearly contain the same classic virulence factors for that species. Of course, in the end such annotations should always be thought of as an initial guide, or hypothesis-generating for more in depth future analysis, due to the highly contextual nature of virulence.

## INCOMPLETE GENOME ANALYSIS

Previously IslandViewer was limited to predicting GIs in only complete, annotated genomes. While this was valuable, and widely utilized by those wanting to analyze their completely sequenced new genome, or wishing to analyse a complete genome that was public but not yet in the IslandViewer pre-computed dataset, one of the most requested features for IslandViewer has been the ability to analyse incomplete genomes. Consistent with this, one the most frequent errors detected on the site was also due to users trying to upload sequence contigs for analysis that were not correctly concatenated together, in an attempt to analyse an incomplete genome. This high demand for analysis of incomplete genomes is in part due to the lack of GI predictors that can make predictions for incomplete genomes. The only GI predictor currently capable of making predictions in incomplete genomes is GI-POP (18), but the web server has been unavailable since publication. The ability to analyse GIs in incomplete genomes has now been incorporated into IslandViewer 3, using a straight forward approach. Annotated sequence contigs must be provided, and a completed microbial genome must be selected to use as a reference to align the contigs. The uploaded contigs are aligned against the user-selected reference genome using the Mauve contig mover (19), and then a single concatenated sequence is generated based on this alignment. In cases where the reverse complement of a contig is aligned to the reference sequence, the reverse complement of that contig is included in the concatenated sequence instead of the original contig sequence. Any unaligned contigs are included at the end of the concatenated sequence for analysis, but they are clearly labeled as unaligned contigs in the IslandViewer output. This ‘concatenated-contigs genome’ is then run through the existing IslandViewer pipeline. This simple approach allows for the identification of GI predictions in draft genomes, with all its caveats. Cautionary points are therefore mentioned on the IslandViewer 3 website associated with this type of analysis. However, it should be noted that while there are limitations to GI analysis of such draft or incomplete genomes, reasonable results can be obtained for genomes with few contigs that aligned to a highly similar reference genome such as from a closely related strain (this observation is based on a comparison of GI predictions for incomplete versus complete versions of the same exact genome; Shay and Brinkman, unpublished). Clearly a more in depth analysis of the accuracy of incomplete genome GI predictions is required in the future for different kinds of predictive scenarios, and we make this clear on the website. However, at minimum, providing a simple concatenated genome capability, of the type users strongly requested who are analyzing highly similar isolates of a strain, seems reasonable, given that users were essentially widely doing this but would benefit from an easier upload tool. Finally, note that in the resulting IslandViewer 3 output for an incomplete/draft genome, contig boundaries are denoted by grey jagged lines to ensure users are aware of when a GI prediction is close to a contig end, and any unaligned contigs are also shown in grey in the alignment plot. Again, as noted on the website, this tool should really only be used at this time for incomplete genomes with very few contigs, that are highly similar to other isolates of the same species.

## OTHER BACKEND UPGRADES

Earlier versions of IslandViewer were built upon MicrobeDB (20), a database of all complete bacterial genomes as downloaded from NCBI (ftp://ftp.ncbi.nih.gov/genomes/Bacteria/). As the number of genomes continues to grow, the coupled IslandViewer and MicrobeDB database became very inefficient. IslandViewer 3 has now been completely separated from MicrobeDB and only stores information relevant to IslandViewer users. At the time of publication, pre-computed analysis of 2794 genomes are now available through IslandViewer 3. Pre-computed analyses will be updated monthly to include new completed bacterial or archaeal genomes from the NCBI upon further upgrading our backend update scripts to reflect changes in file structure and genome annotations at the NCBI. In addition to this, IslandViewer 3 can now submit custom analyses on a new set of powerful cluster nodes with a robust queuing system. This will improve processing time of custom analyses of existing genomes (for example, using different IslandPick comparative genomics criteria) and analysis of new user-uploaded genomes, and will virtually eliminate the most common errors prevalent in the former system.

## DISCUSSION AND CONCLUDING REMARKS

Overall, IslandViewer 3 presents innovative analysis and visualization tools to not only enhance the study of GIs, but to also generally provide an interactive genome visualization experience for all complete bacterial and archaeal genomes. The new interface can be used to search for genes of interest, select or zoom in on features, and generally navigate through a genome to understand the virulence factor and antimicrobial resistance gene content while simultaneously evaluating GI predictions, as highlighted in Figure 1. This figure also acts as a reminder that sequence composition-based methods, such as IslandPath-DIMOB and SIGI-HMM, do not predict island boundaries, but rather predict GI regions that IslandPick, or more manual analysis, can refine in terms of specific island border determinations. IslandPick is particularly useful for custom analysis of genomes, for example, identifying more recently introduced GIs in an infectious disease outbreak isolate's genome, versus a type strain. The more flexible, interactive genome viewer now in IslandViewer 3, with its zooming features, will greatly facilitate more refined analysis of such outbreak isolates. The expanded virulence, resistance and pathogen-associated gene annotations are also important in enabling further analysis of pre-computed genomes in the context of richer annotation. This is valuable not just for GI prediction, but may prove valuable for more general investigations of bacterial or archaeal genomes. However, GI prediction is still likely to remain a key analysis for many new microbial genomes, as our understanding increases of the important role GIs have in microbial evolution and the acquisition of new adaptations of medical and other interest.
